# Lessons learned about teaching and medical care at the Instituto da Criança e do Adolescente do Hospital das Clínicas da Faculdade de Medicina da USP during the COVID-19 pandemic

**DOI:** 10.6061/clinics/2021/e3579

**Published:** 2021-11-23

**Authors:** Ana Cristina Aoun Tannuri, Ana Maria de Ulhôa Escobar, Filumena Maria da Silva Gomes, Uenis Tannuri, Sandra Josefina Ferraz Ellero Grisi

**Affiliations:** Departamento de Pediatria, Faculdade de Medicina FMUSP, Universidade de Sao Paulo, São Paulo, SP, BR.

The restrictive measures imposed on the world to control the coronavirus disease 2019 (COVID-19) pandemic had a significant impact on all spheres of society (economic, social, and behavioral). In particular, education had to undergo a series of adaptations in an attempt to reduce the losses arising from the lack of contact and face-to-face relationships. Never has it been so difficult to practice this famous quote by Malcolm Knowles: “We will learn no matter what! Learning is as natural as rest or play. With or without books, inspiring trainers, or classrooms, we will manage to learn” (1). From elementary to graduate school, different forms of distant interaction had to be implemented on an emergency basis.

The first resource used for teaching at all levels during the pandemic was online teaching, the challenges of which impact both those who teach and those who learn. Teachers often do not adequately deal with the diverse meeting platforms available, particularly those teachers who have to learn how to use such technology in adulthood and adapt to the routine of administering online tasks. Simultaneously, students also struggle with their own issues, such as sustaining attention for a long period, absorbing the content delivered in a new fashion, and spending multiple hours in front of the computer. Technical challenges such as problems with audio and video, downloading or streaming errors, login problems, poor internet quality, and security issues must be considered. However, it is also known that online teaching encourages student autonomy and protagonism.

Medical education in undergraduate and graduate programs deserves special attention. The unique integration of the theoretical framework with the practice of anamnesis and physical examination of the patient, associated with the diversity of presentations of each clinical case, makes the practice of medicine, as well as its teaching, particularly complex. Thus, one of the challenges posed by the pandemic was the need to maintain the training of students and physicians in this context.

The medical course curriculum of Faculdade de Medicina da Universidade de São Paulo (FMUSP) underwent extensive restructuring seven years ago, aiming at the integration and better contextualization of the taught subjects. In pediatric teaching, interdepartmental classes were implemented in the form of curricular units such as Life Cycle I, II, and III, and Integration of Major Pediatric Diseases and Situations, and no longer in departmental subjects. Theoretical classes also incorporated practicals in the teaching of clinical pediatrics. The course exams were in person, but online, with laptops in the classroom. Due to this recent change, adaptation to the online teaching environment was even more difficult as the institution was still transitioning to this new curriculum. A good solution was the interaction of two main formats of activities: asynchronous distance education, such as recorded videos and podcasts, and synchronous (live) distance education (SDE), such as video conferences and virtual classrooms. The undeniable advantages of recorded classes are noticeable for both the faculty and student body. For the former, there are considerable savings in time and resources because once the lesson is recorded, it can be made available several times to different classes without prejudice to its content. For the students, the possibility of watching the recorded lessons more than once, pausing whenever necessary, and accessing the lessons at the most convenient time are important advantages. However, there is a consensus that such activities should not last more than 40 to 50 minutes.

The SDE format, in which teachers and students connect at the same time on the virtual meeting platform, allows for greater interaction, student participation, and elaboration of questions and answers. Such activities, mainly discussions of clinical cases in small groups, allow the strengthening of the knowledge previously learned in recorded classes as well as the exercising of clinical reasoning and clarification of any doubts. Such meetings also allow teachers to control student attendance and performance in both the pre- and posttests.

An additional challenge was the application of tests or other forms of measuring skills and acquired knowledge. Different mechanisms were developed to achieve a relatively fair way to assess students’ learning, such as blocking other websites during tests, defining a specific duration for completion, and designing questions based on clinical reasoning rather than mere content memorization.

Practical teaching during the hospital internship for the 5^th^ and 6^th^ year students became unviable in the first months of the pandemic, in which Hospital das Clínicas was completely restructured for the care of patients with COVID-19 from all over the state. However, the impact of the pandemic on pediatrics, and consequently, in the teaching of students, was somewhat different. Symptoms of SARS-CoV-2 infection in children are mostly mild (2). In addition, isolation measures also caused a significant decrease in the incidence of contagious respiratory diseases, which are highly prevalent in the pediatric age group, especially during fall and winter. Thus, there was a drastic decrease in Emergency Room visits and hospital admissions for children.

Despite this, the Pediatric Department at the Hospital Universitário (HU) and the Instituto da Criança (ICr) made significant efforts to overcome possible gaps in student learning through additional activities and online discussions. Students were vaccinated against SARS-CoV-2 and performed internships in emergency rooms and infirmaries, with rotations in small groups.

Theoretical classes were performed remotely, synchronously, and were available in the classroom. Situations involving telesimulation were implemented, and a defined period was given for students to solve tasks. Team-based learning (TBL) is a structured form of small-group learning that emphasizes student preparation out of class and the application of knowledge in class. Students were organized strategically into diverse teams of five to seven that work together throughout the class. Platforms that allow the main student group to be broken down into smaller groups were successfully used to enable online TBL. Even essentially practical activities such as Objective Structured Clinical Examination could be transferred to the virtual environment. With the decrease in the number of consultations in the Emergency Room (mainly in HU), and in the number of hospitalized children, the skills laboratories at the HU and ICr started to be used.

However, the internships in neonatology at HU and ICr (which include emergency care and rear wards) could be maintained with excellent structure and the same duty schedule as both the number of births in HU and the emergency care of children in ICr (where most patients present comorbidities) could be maintained despite the pandemic.

In ICr, professionals were shifted, and the physical space was readjusted to care for children suspected and unsuspected of COVID-19 infection. Thus, screening and differential input flows, collection structure, and beds (infirmary and intensive care unit) were installed for children with or without flu symptoms. All newborns from the neonatal intensive care unit of the Central Institute as well as the equipment and professionals of this unit were transferred to the HU. The tele-assistance system was implemented to re-evaluate patients after discharge from the Emergency Room and in all specialty clinics (more than a thousand teleconsultations have been conducted since then).

During 2020, this restructuring allowed 145,391 outpatient consultations, 8,041 Emergency Room visits, and 6,817 hospitalizations to be performed at the ICr.

The pediatric surgery and liver transplantation divisions are characterized by a diversity of pathologies, ranging from low complexity cases to major surgeries, such as correction of severe congenital digestive and respiratory diseases, resections of large tumors, and a robust liver transplantation program. Initially, it was decided to suspend all elective procedures, including liver transplants due to chronic diseases. However, the treatment of a large number of children with complicated surgical and oncological conditions was considered critical and therefore could not be interrupted.

After the first two months of the pandemic, with a better understanding of the transmission route and with the possibility of a more adequate control of COVID-19, we returned to performing liver transplantation with cadaveric donors and semi-elective surgeries. We then returned to performing living donor liver transplants, strictly following all safety protocols to avoid contagion of the recipient and, above all, of the living donors. No increased morbidity or complications were observed in comparison with previous periods.

In the period between the onset of the pandemic in Brazil (February 2020) and the present time (September 2021), 82 liver transplants were performed in the institution, 58 of which were from living related donors and 24 were from cadaveric donors, with a survival of 76 patients to date (92.7%). There were no cases of COVID-19 in the perioperative period, either in living donors or recipients (3).

Currently, with vaccinations evolving at a faster pace, there have been glimpses of good prospects. The extensive clinical observation and the results of some scientific studies already allow us to trust the efficacy of vaccines as protecting, if not against contagion, then at least against the progression to severe forms of the disease, even with the emergence of new variants of viral strains. With this, we can schedule a gradual and conscientious return of didactic activities in person. Initially, a mixture of distance theoretical classes with face-to-face practical activities will be designed, with emphasis on student contact with patients, as well as activities of an essentially practical nature.

Certainly, some of the behavioral and social changes imposed by the pandemic will certainly remain, even when the pandemic ceases to exist. Currently, within the scope of the FMUSP post-graduation program, qualification exams and thesis defenses are clear examples of how the adaptation to the online learning environment can have lasting positive impacts. Such activities fit perfectly in this format and allow members of these examining boards, from other cities and states (even countries), to participate without the financial cost and time required for their travel. The number of students enrolled in online postgraduate courses at the Pediatric Department has increased considerably, and this has increased exchange with the students and faculties of other disciplines, such as psychology and physical education.

In the end, we can also presume that many graduation programs (including ours) will assume a hybrid aspect, combining “online” activities, such as theoretical classes, with face-to-face practical activities. Even though in-person interaction is paramount in medical training, the road ahead will be paved with hybrid formats that include online resources.
